# Folate status of gut microbiome affects *Caenorhabditis elegans *lifespan

**DOI:** 10.1186/1741-7007-10-66

**Published:** 2012-07-31

**Authors:** Theresa PT Nguyen, Catherine F Clarke

**Affiliations:** 1 UCLA Department of Chemistry and Biochemistry and the Molecular Biology Institute, 607 Charles E Young Dr E, Los Angeles, CA 90095 USA

## Abstract

In a paper in *BMC Biology *Virk *et al*. show that *Caenorhabditis elegans *lifespan is extended in response to a diet of folate-deficient *Escherichia coli*. The deficiencies in folate biosynthesis were due to an *aroD *mutation, or treatment of *E. coli *with sulfa drugs, which are mimics of the folate precursor *para*-aminobenzoic acid. This study suggests that pharmacological manipulation of the gut microbiome folate status may be a viable approach to slow animal aging, and raises questions about folate supplementation.

See research article http://www.http://www.biomedcentral.com/1741-7007/10/67

## Aging in *Caenorhabditis elegans *- nature versus nuture (or a diet of *E. coli*)?

The first genes extending lifespan were identified in the nematode *Caenorhabditis elegans *[1], and several of the metabolic pathways they are involved in are conserved in other species, including flies, mice and humans. Some of the pioneering studies on aging in *C. elegans *made use of RNA interference (RNAi) to manipulate gene expression, a strategy that is easily applied in *C. elegans *by feeding the worms on *Escherichia coli *strains expressing the relevant RNA sequence.

**Figure 1:**
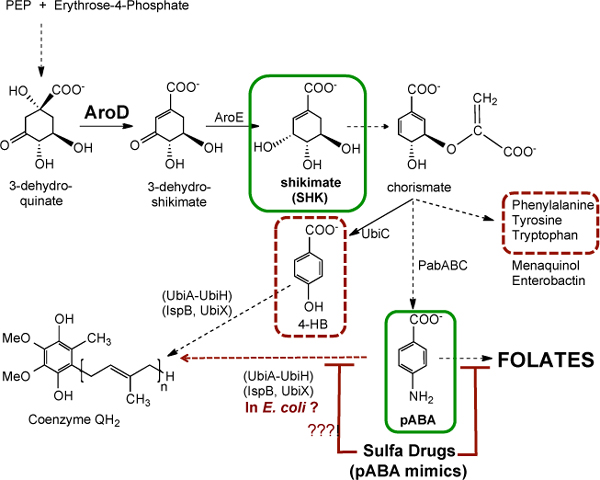
***E. coli aroD *mutants have defects in shikimate biosynthesis and fail to produce diverse aromatic metabolites**. An abbreviated sequence of reactions involved in the *E. coli *biosynthesis of shikimate (SHK) and chorismate, and the products of this metabolism, are indicated. Black solid arrows indicate one step; black dashed arrows indicate multi-step pathways (four gene products catalyzing three steps convert SHK to chorismate). Chorismate is a precursor in the synthesis of aromatic amino acids (Phe, Tyr, Trp), menaquinol (used in *E. coli *respiratory electron transport and a precursor of vitamin K), enterobactin (a siderophore used by *E. coli *to acquire iron), 4-hydroxybenzoic acid (4-HB; a precursor of coenzyme Q) and *para*-aminobenzoic acid (pABA; a precursor of folate). Addition of either SHK or pABA reversed the lifespan extension in *C. elegans *fed a diet of *aroD *mutant *E. coli *(green boxes). In contrast, addition of the aromatic amino acids or 4-HB failed to reverse the lifespan extension (red dashed boxes). pABA also serves as a precursor of coenzyme Q in the yeast *Saccharomyces cerevisiae *[6]. However, it is not yet know whether pABA is an aromatic ring precursor in coenzyme Q biosynthesis in *E. coli *(red dashed arrow with question marks). Sulfa drugs mimic pABA and hence inhibit folate biosynthesis in microbes, but the effect on coenzyme Q has yet to be determined.

However, it is becoming increasingly clear that the *E. coli *diet itself can have profound affects on *C. elegans *lifespan, and the degree of bacterial colonization within the worm gut has been shown to correlate inversely with worm lifespan [2]. In a recent paper in *BMC Biology*, Virk *et al*. [3] capitalize on a serendipitous finding - they show that a *C. elegans *lifespan extension phenotype originally attributed to an RNAi clone targeting the *ugt-27 *gene is actually due to a spontaneous mutation present in the host *E. coli *strain. The authors then use classic nutritional selection experiments and identify the mutation as an IS1 insertion element within the *E. coli aroD *gene. AroD is a dehydratase required for the production of shikimate (SHK; Figure 1), which is in turn a precursor of chorismate, a precursor of a wide variety of aromatic compounds in *E. coli*. Thus, the *aroD *mutation affects production of Phe, Tyr, and Trp (essential aromatic amino acids), menaquinol (vitamin K_2_), enterobactin (involved in *E. coli *iron uptake), coenzyme Q (an essential lipid component of the respiratory chain) and folates (vitamin B9). Virk *et al*. convincingly demonstrate that the lifespan extension in *C. elegans *can be returned to normal when the diet of *E. coli aroD*^- ^is supplemented with either SHK or the folate precursor, pABA (Figure 1), but not when it is supplemented with the other aromatic products of this pathway. Because pABA supplementation abrogates the lifespan extension of *C. elegans *fed the *aroD*^- ^*E. coli *diet, Virk *et al*. focus their attention on folate metabolism.

## pABA and sulfa drugs - do they impact more than just folate metabolism?

**Figure 2:**
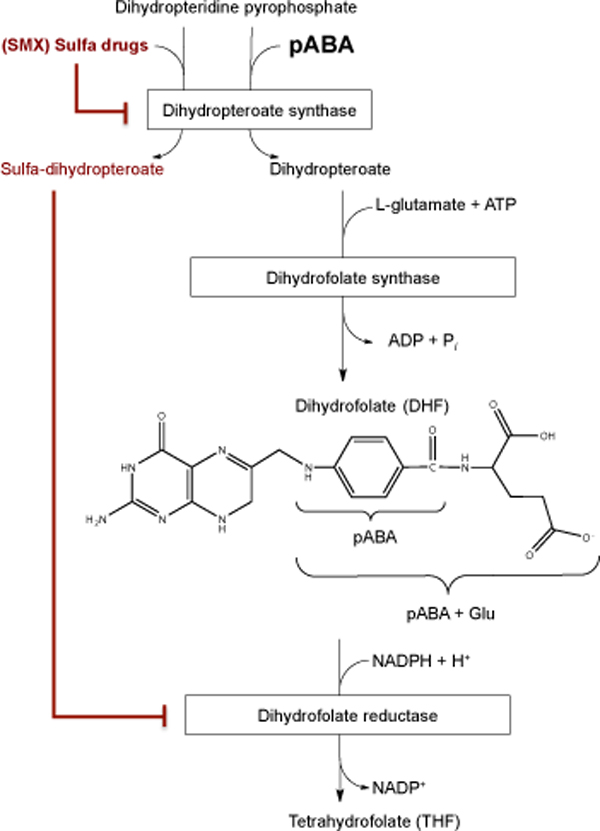
**Sulfa drugs strike more than once in the folate biosynthetic pathway**. Dihydropteroate synthase catalyzes the condensation of dihydropterin pyrophosphate and pABA to form dihydropteroate. Glutamate chain length depends on the cellular role of the folate derivative, and the final tetrahydrofolate product may have from one to six Glu residues. As indicated by the red lines, sulfa drugs (such as sulfamethoxazole, SMX) block folate production at the dihydropteroate synthase step by competing with pABA, and by acting as a substrate itself. The resulting sulfa-DHP derivative is an additional folate biosynthesis inhibitor, competing against dihydrofolate (DHF) for reduction by dihydrofolate reductase. *E. coli *are capable of transporting and hydrolyzing pABA-glu (a folate breakdown product) to produce free pABA and glutamate for *de novo *folate synthesis [4].

In *E. coli *and other microbes, pABA is a precursor of dihydrofolate (DHF; Figure 2). Folates play crucial roles in metabolism of amino acids, purines and pyrimidines. Reduction of DHF produces the active form of the vitamin, tetrahydrofolate (THF), which in turn is a versatile one-carbon carrier that functions to donate and accept one carbon units at differing states of oxidation, including 5-methyl-THF, 5,10-methylene THF, 5,10-methenyl-THF, and formyl-THF. These folate coenzymes play essential roles in the synthesis of nucleic acids and amino acid metabolism. Because folates also contain variable numbers of glutamate residues, cells contain a truly bewildering array of mono- and poly-glutamated folate derivatives. Formyl-THF-Glu_3 _is the most abundant species in *E. coli*, and the authors show its content is severely decreased in the *E. coli aroD *mutant. Furthermore, they show that sulfamethoxazole (SMX), a sulfa-drug antibiotic and well-known pABA mimic, elicits a dose-dependent lifespan extension when added to *C. elegans *growth medium containing the standard laboratory *E. coli *OP50 diet. The authors show that formyl-THF-Glu_3 _content is also dramatically decreased in SMX-treated OP50 *E. coli*, and in *C. elegans *fed the SMX-treated OP50 *E. coli *diet.

The results show that inhibition of folate synthesis in the microbe extends *C. elegans *lifespan. Is a decreased supply of folate *per se *mediating lifespan extension? Virk *et al*. tested whether the direct addition of folate to the *aroD E. coli *diet reversed *C. elegans *lifespan extension. At the outset, this seemed like a straightforward experiment: *E. coli *(and other microbes) do not assimilate exogenous folates (this is why the sulfa drugs are so effective). However, the results obtained showed intermediate effects. Folate supplementation only partially reversed the lifespan extension. In fact, the authors showed that this folate supplementation experiment is not so simple. When the *E. coli aroD *mutant was grown in the presence of exogenous folate the formyl-THF-Glu_3 _content of the bacteria increased! While microbial folate catabolism pathways are not completely understood, it is clear that *E. coli *has a transporter that allows for uptake of pABA-glutamate, a folate breakdown product (Figure 2), and a hydrolase that can process pABA-glu into free pABA and glutamate [4]. Hence, the catabolism of exogenously added folate by *E. coli *(and by the gut microbiome) leads to *de novo *synthesis of more folate. Finally, the extent to which *C. elegans *takes up exogenously added folate was not determined, although this may have important implications for whether folate supplementation is an effective way to boost folate stores in *C. elegans*.

So the jury is still out - clearly the microbe's folate status affects *C. elegans *lifespan. But whether this is due to the content of folate in the diet, or to indirect effects of the folate status on *E. coli *or *C. elegans *is still an open question. It would be interesting to test folate supplementation with axenic medium, where *E. coli *is eliminated from the *C. elegans *diet and the folate content could be precisely defined [5]. It would also be important to determine whether the enhanced lifespan of *C. elegans *fed either the *E. coli aroD *mutant, or OP50 treated with SMX, is related to the extent of bacterial colonization of the worm intestine [3].

It is intriguing to consider other possible fates of pABA in this model, as addition of pABA did completely reverse the lifespan extension in *C. elegans *fed the *aroD*^- ^diet. In the yeast *Saccharomyces cerevisiae*, pABA serves as an alternative aromatic ring precursor in the biosynthesis of coenzyme Q [6]. The fate of pABA as a ring precursor of coenzyme Q in *E. coli *or *C. elegans *is still uncertain. Hence, it would be of interest to monitor the effect of 4-HB or pABA supplementation on the coenzyme Q content in *E. coli aroD *mutants. Virk *et al*. showed that addition of 4-HB, an established ring precursor of coenzyme Q in *E. coli*, did not reverse the lifespan extension. Nonetheless, since *C. elegans *fed respiratory defective mutant strains of *E. coli *diet show an extended lifespan [7], the possible impact of SMX and pABA on coenzyme Q content and respiratory metabolism in *E. coli *remains an intriguing avenue of future investigation.

Not only do sulfa drugs directly inhibit folate biosynthesis through competition of SMX with pABA at the dihydropteroate synthase step, but they also produce sulfa-dihydropteroate in the process, and this in turn acts as an inhibitor of dihydrofolate reductase (DHFR), the final step in the production of THF [8]. Although sulfa-dihydropteroate is excreted by *E. coli*, it is likely to be present in the worm gut. This raises the question of whether other drugs (such as methotrexate, an inhibitor of dihydrofolate reductase and widely used in chemotherapy) might also impart lifespan extension effects when added to the worm diet.

## Folate supplementation, sulfa drugs, and human aging

Like *C. elegans*, mammals are unable to synthesize folate and acquire the metabolite through diet and gut microflora production. Since 1998, the US Food and Drug Administration has required folate supplementation in all cereal grains, which has resulted in higher blood folate content of the adult, non-supplement using population [9]. Recently, a study on the gut microflora of 531 human subjects across a wide range of ages, ethnicities, and geography showed that microbes residing in babies are enriched in genes involved in *de novo *folate biosynthesis, whereas the microbes residing in adult subjects were enriched in genes that metabolize dietary folate and THF [10]. However, because folate supplementation regulations and diet differ in the sampling population, there is insufficient data to assess whether the changes in microbial folate biosynthesis gene expression are linked to dietary folate. Interestingly, Virk *et al*. note that sulfa drugs have been reported to inhibit microbiome folate synthesis and extend lifespan in rats [1]. While the mechanism remains to be determined regarding how genetic or pharmacological knockdown of folate in *E. coli *can enhance *C. elegans *lifespan, Virk *et al*. have raised the intriguing possibility that manipulation of the folate status of gut microflora may impact lifespan in other species.
